# Pharmacology, Toxicology, and Therapeutics of Minerals in Traditional Medicine 2021

**DOI:** 10.1155/2023/9823746

**Published:** 2023-09-20

**Authors:** Jie Liu, Uma Maheswari Krishnan, Lixin Wei

**Affiliations:** ^1^Key Lab for Basic Pharmacology of Ministry of Education and Joint International Research Laboratory of Ethnomedicine, Zunyi Medical University, Zunyi, China; ^2^Centre for Nanotechnology & Advanced Biomaterials, School of Chemical & Biotechnology, SASTRA Deemed University, Thanjavur 613 401, Tamil Nadu, India; ^3^Qinghai Key Lab of Tibetan Medicine Pharmacology and Safety Evaluation, Northwest Institute of Plateau Biology, Chinese Academy of Sciences, Xining 810008, China

Minerals have been used in traditional medicine since ancient times and are still used in Indian Ayurveda and Chinese medicines including Tibetan medicine [1–4]. The processing/preparation procedures distinguish minerals used in traditional medicines from environmental toxic metals such as mercury-based Ayurveda [1], Zuotai, and various Bhasmas [3]. Minerals are not used alone but as polyherbal-metallic preparations for oral administration [1, 2, 4]. The aim of this Special Issue is to collate original research and review articles dealing with minerals in traditional medicines.

A review paper [5] summarized 10 herbo-mineral preparations used in Sri Lanka regarding the purification, detoxification, or incineration techniques. The minerals included in the 10 traditional medicines are copper sulphate, aluminum sulphate, borax powder, sulphur, cinnabar (HgS), realgar (As_2_S_2_), orpiment (As_2_O_3_), ammonium chloride, magnesium silicate, zinc oxide, and mercury (*Parada*). It should be noted that only sulfide and oxide forms of metals are used in oral traditional medicines, and these detailed methods highlight the importance of mineral processing techniques before addition to traditional medicines. Processing gypsum could change its mineral properties and biological activities when used in traditional medicines [6]. The preparations of minerals used in traditional medicines such as Zuotai and Bhasmas are very important and are needed for quality control [1, 3, 7, 8].

Quality control is an important issue for mineral-containing/contaminated traditional medicines. In this regard, Xiao et al. [9] used HPLC-ICP-MS to detect arsenic content, speciation, and distribution in wild *Cordyceps sinensis*, a famous traditional medicine, to provide scientific basis of quality control. Fu et al. [10] used ICP-MS to detect 18 element contents across 10 batches of Qishiwei Zhenzhu pills (QSW) produced by 5 pharmaceuticals. QSW is a famous Tibetan medicine listed in Pharmacopeia of China for many diseases. To establish a quality control for toxic elements (Hg, Pb, As, and Cd), essential elements with potential toxicity (Cu, Mn, Cr, and Co) and elements with medicinal use (Li and Au) could be used to ensure its efficacy and reduce adverse effects and toxicity.

Oral administration is the main route for using herbo-mineral preparations. Song et al. [11] determined 18 elements in QSW for absorption and distribution to the liver, kidney, and brain and excretion by feces, urine, and hair in rats with middle cerebral artery occlusion (MACO). The majority of minerals were excreted by feces implying the long-term stay of elements in the gut that could affect the gut-microbiota-brain axis in QSW-induced protective effects against cerebral ischemia-reperfusion injury [12].

QSW is effective against cerebral ischemia stroke in animal models. Mechanistic studies revealed QSW indeed affected gut microbiota in rats with MACO. At the phylum level, it can regulate the abundance of Firmicutes and Proteobacteria; at the genus level, it can adjust the abundance of *Escherichia* and *Shigella*; and at the species level, it can adjust the abundance of *Lactobacillus johnsonii* and *Lactobacillus reuteri*. QSW can also decrease inflammatory factor IL-1*β*, TNF-*α*, and IL-6 expression in the hippocampus of MACO rats, thus reducing oxidative damage caused by ischemia and reperfusion [12]. Cerebral ischemia injury can also be alleviated by cinnabar (HgS) and realgar (As_4_S_4_) containing An-Gong-Niu-Huang Wan (AGNH), and cinnabar and realgar are shown to be essential ingredients in the recipe [13]. The entire AGNH formulae can also protect against the hepatorenal toxicity produced by cinnabar and realgar alone and reduce Hg and As accumulation in tissues, implying herbal constituents in polyherbo-mineral preparations could offset the toxic effects of minerals [14, 15].

New approaches are attempted to explore the pharmacological basis of herbo-mineral preparations. The mechanism of arsenic trioxide against hepatocellular carcinoma (HCC) was examined through network pharmacology, and various pathways including TNF signaling pathway, AMPK signaling pathway, NF-kappa B signaling pathway, and several targets-pathways-HCC network molecules could be potential molecular targets for arsenic against HCC [16]. Modulation of gut microbiota could be an important target for QSW [12] to produce neuroprotective effects but also for Hua-Feng-Dan to produce neuroprotective effects [17] fortifying the role of metal composition and herbal interactions in herbo-mineral preparations. RNA-Seq technology was also employed to analyze the adaptive mechanism of hepatoprotection with cinnabar and realgar-containing Hua-Feng-Dan [18] and antihepatic fibrotic mechanism with Ganxianfang formula [19].

Thus, with contributions from investigators from different countries, this Special Issue presented recent experimental findings and reviews on pharmacology, toxicology, and therapeutics of minerals in traditional medicines. In this Special Issue, there are more valuable manuscripts besides those given above. We hope the readers will be interested in approaching minerals in traditional medicines from “processing,” “formulae,” quality control, pharmacokinetics, pharmacology, toxicology, and bioinformatics.

*Jie Liu* *Uma Maheswari Krishnan* *Lixin Wei*
